# Amputation at the Hip-Joint

**Published:** 1871-04

**Authors:** 


Amputation at the Hip-Joint.—Dr. Edwin Powell reports a case of this operation for chronic inflammation of the hip and knee-joints, with recovery. The patient had been for two years a sufferer from the affection of the joints, which, with occasional periods of improvement, had grown steadily worse, so that at the time of operation he was much emaciated, and was compelled to take ten grains of morphine daily to get relief.

The antero-posterior-flap operation was the one resorted to; the shock was very great, yet after reaction the patient began to improve and went on to complete recovery, so far as the amputation was concerned.

An attack from phthisis pulmonalis supervened, from which the patient died ten months after the operation.—*Transactions Ill. State Medical Society.*

